# Circulating tumor cells and γH2AX as biomarkers for responsiveness to radium-223 in advanced prostate cancer patients

**DOI:** 10.2144/fsoa-2019-0092

**Published:** 2019-12-05

**Authors:** Jonathan Chatzkel, Jesse Mocha, Johnna Smith, Jun-Min Zhou, Youngchul Kim, Ghassan El-Haddad, Jingsong Zhang

**Affiliations:** 1 Division of Hematology & Oncology, University of Florida, Gainesville 32608, FL, USA; 2 Department of Genitourinary Oncology, H. Lee Moffitt Cancer Center & Research Institute, Tampa 33612, FL, USA; 3 Department of Diagnostic Imaging & Interventional Radiology, H. Lee Moffitt Cancer Center & Research Institute, Tampa 33612, FL, USA; 4 Department of Immunology, H. Lee Moffitt Cancer Center & Research Institute, Tampa 33612, FL, USA; 5 Cancer Biology & Evolution Program, H. Lee Moffitt Cancer Center & Research Institute, Tampa 33612, FL, USA

**Keywords:** biomarkers, circulating tumor cells, homologous repair deficiency, metastatic castrate-resistant prostate cancer, radium-223, γH2AX

## Abstract

**Aim:**

Radium-223 improves overall survival in patients with metastatic castration-resistant prostate cancer to the bone. Radium-223 causes double-strand DNA breaks and produces γH2AX, a potential biomarker for response. We examined the feasibility of tracking γH2AX positivity and numeration in circulating tumor cells.

**Patients & methods:**

Ten patients with biopsy-confirmed symptomatic M1b castration-resistant prostate cancer received radium-223 as standard of care and were assessed for γH2AX level changes following doses 1, 3 and 6.

**Results:**

Trend tests confirmed that patients with ≥50% increase in circulating tumor cells positive for γH2AX postradium-223 therapy had a lower risk of death (p = 0.035).

**Conclusion:**

Regular interval measurements of γH2AX are feasible. The potential correlation between γH2AX changes and overall survival warrants further investigation.

## Lay abstract

Radium-223 is used to treat patients with prostate cancer that has spread to the bone. This therapy causes a type of DNA damage that leads to the release of a protein called γH2AX. We hypothesized that measuring this protein in the blood after treatments with radium-223 was feasible and could help to determine how well patients responded to therapy. Our trial enrolled ten patients and suggested that increases in γH2AX after therapy may be associated with longer survival. The correlation warrants further investigation.

Prostate cancer affects more than 160,000 men in the USA each year and kills more than 26,000 [1]. Although androgen-deprivation therapy is an effective first line of treatment for patients with advanced prostate cancer, most patients eventually develop castrate-resistant disease and require additional therapies [2]. The ALSYMPCA trial demonstrated that radium-223, a targeted α emitter, improved overall survival (OS) compared with placebo among patients with symptomatic metastatic castration-resistant prostate cancer (mCRPC) to the bone [3]. Despite this improvement in OS, only 16% of treated patients in the trial had a ≥30% decline in prostate-specific antigens (PSAs) at week 12. An exploratory analysis of the ALSYMPCA trial reported a correlation between total alkaline phosphatase (tALKP) and LDH decline at week 12 and longer OS [4]. However, these correlations did not meet statistical surrogacy requirements. A retrospective analysis of 92 mCRPC patients treated with radium-223 reported the potential prognostic value of baseline clinical variables, including Eastern Cooperative Oncology Group performance status, hemoglobin <12 g/dl and PSA >20 ng/ml [5]. In another retrospective study, which included 64 mCRPC patients, several commonly used clinical variables were identified by multivariate analyses to be significantly associated with survival benefit. These clinical variables included no prior chemotherapy, ≤5 bone metastases, baseline ALKP <115 U/l and tALKP response after radium-223 treatment [6]. However, as demonstrated by the exploratory analyses of the ALSYMPCA trial, these commonly used clinical variables alone are unlikely to be robust enough to predict the survival benefit from radium-223 [4].

The measurement of circulating tumor cells (CTCs) has been studied as a prognostic marker in a variety of cancers [7–10]. CellSearch is a CTC assay that was originally developed by Veridex LLC and has been validated and approved by the US FDA for clinical use. CTCs isolated from patients with mCRPC have demonstrated features consistent with those of prostate cancer, including the expression of PSA, *AMACR* and prostate-specific gene abnormalities; these include androgen receptor copy number amplifications, phosphatase and tensin homolog deletions and *TMPRSS2*:*ERG* fusion products [11,12]. Several studies have demonstrated the prognostic value of CTCs for patients with prostate cancer [9,13–16]. In addition, among mCRPC patients starting first-line chemotherapy, post-treatment CTCs were stronger predictors of OS than a 50% decline in PSA [14]. A recent study showed that patients with ≤5 CTCs at baseline were more likely to complete a 6-treatment course of radium-223 and had significantly longer OS [17].

As a targeted α-emitter, radium-223 induces primarily double-strand breaks in the DNA of cancer cells [18,19]. γH2AX is produced when these breaks occur and allows for the recruitment of proteins involved in DNA repair and chromatin remodeling [20–22]. The formation of γH2AX can be detected via immunofluorescence, as detailed in Figure 1 [20,23]. We developed an assay to detect γH2AX in prostate cancer CTCs and assess the feasibility of tracking changes in CTC γH2AX positivity and numeration before doses 1, 3 and 6 of radium-223. This prospective biomarker pilot study included ten mCRPC patients. We hypothesized that γH2AX was a useful biomarker for patient responses to radium-223 and that tumor-cell damage induced by radium-223 therapy would increase the proportion of CTCs positive for γH2AX. To our knowledge, the use of γH2AX as a biomarker for mCRPC has not yet been evaluated.

**Figure 1.**
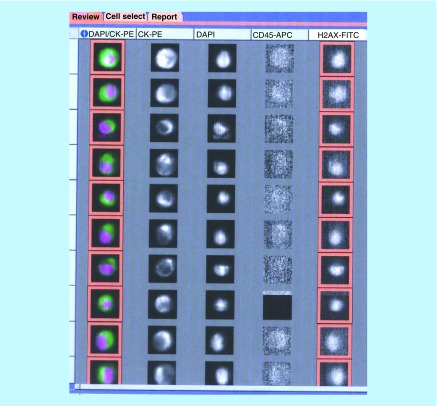
Detection of γH2AX in DU145 prostate cancer cells treated with topoisomerase 1 inhibitor SN38. DU145 cell lines were treated with 0.1 μM of SN38 for 8 h before being spiked into human healthy donor blood. γH2AX fluorescein isothiocyanate was applied to the open channel. DU145 cell lines were identified via positive DAPI, positive CK-PE and negative CD45. Approximately 40% of SN38-treated DU145 cells were positive for γH2AX. CK–PE: Cytokeratin–phycoerythrin; DAPI: 4’, 6-diamidino-2-phenylindole; FITC: Fluorescein isothiocyanate.

## Materials & methods

### Patients

The study enrolled patients with biopsy-confirmed prostate cancer who were receiving radium-223 as standard of care for symptomatic M1b CRPC that was identified by either a bone scan or NaF positron emission tomography imaging. Patients were also required to have at least two CTCs at baseline; to be on either a gonadotropin-releasing hormone analog or to have received surgical castration; to have an Eastern Cooperative Oncology Group score of 0 to 2 (scores of 3 were acceptable if due solely to pain); and to be in stable medical condition with a life expectancy of at least 6 months [24]. Patients were further required to have acceptable laboratory parameters as defined by hemoglobin levels more than 10 g/dl, a platelet count more than 100,000 per μl, an absolute neutrophil count more than 1500 per mm^3^, ALT and AAT levels less than 2.5× the upper limit of normal, total bilirubin levels less than 1.5× the upper limit of normal and creatinine clearance more than 40 ml/min.

Exclusion criteria included exposure to radioisotope therapy within 24 months, exposure to external beam radiation within 12 weeks of the first dose of radium-223, New York Heart Association class III or IV heart failure and the presence of a second malignancy (except nonmelanoma skin cancer or carcinoma *in situ*). The trial was reviewed by the H. Lee Moffitt Cancer Center and Research Institution Scientific and Institutional Review Boards.

### CTC collection, isolation, numeration & the γH2AX assay

For each enrolled subject, 7.5 ml of peripheral blood was collected before and within 96 h after the first, third and sixth doses of radium-223. The blood samples were shipped to Jansen Diagnostics for CTC numeration and γH2AX assays. These assays separated CTCs by using magnetic beads conjugated with antibodies to epithelial cell adhesion molecules. The cells were then stained with anticytokeratin antibodies to identify epithelial cells, DAPI to highlight nuclei, anti-CD45 to exclude contaminating leukocytes and anti-γH2AX antibodies. Cells that were positive for cytokeratin and DAPI but negative for CD45 were selected as CTCs [25]. Total CTCs and γH2AX-positive CTCs were reported and counted. Cell fragments or cells without an intact DAPI nuclear stain were excluded from counting. DU145 prostate cancer cells treated with the DNA-damaging chemotherapy agent irinotecan were pulsed into the peripheral blood of healthy volunteers, which was used to develop the CTC γH2AX assay and serve as positive controls.

### Study design

Eligible patients were enrolled in the study before starting radium-223 therapy as standard of care for their M1b CRPC. The administration of radium-223 was based on the FDA-approved dose and schedule of 1.49 μCi/kg of bodyweight once every 4 weeks. Laboratory testing included a complete blood count and a comprehensive metabolic panel. PSA and testosterone levels were measured at baseline and before each dose of radium-223. Technetium-99m bone scans were performed at baseline and after doses 3 and 6 of radium-223.

The primary objective of the study was to assess changes in circulating prostate cancer cell numeration and changes in CTC γH2AX positivity before and after radium-223 treatment. The secondary objectives included assessing changes in patient-reported pain (as measured by the Brief Pain Inventory) and assessing for a response to therapy as measured via bone scan imaging. Exploratory end points included assessing changes in tALKP phosphatase and PSA. Patients were defined as having a significant increase in γH2AX if their CTCs were at least 20% positive for γH2AX and had a minimum 50% increase in γH2AX-positive CTCs following at least one of the three postradium-223 collections. A decline in tALKP was defined as a ≥30% reduction from the preradium-223 baseline. PSA response required at least a 50% reduction from the baseline PSA. After each radium-223 treatment, patients reported whether their pain had interfered with their general activity over the last 24 h and rated both their average and worst pain levels during this time on a scale from 1 to 10. Patients were defined as having pain progression if their pain had increased above the baseline level for at least two of the three measures. Patients were removed from the study if they developed persistent grade 4 side effects based on the Common Criteria for Adverse Events version 4.0 or if they had disease progression based on both imaging criteria and an assessment of symptoms [26]. Bone scans were assessed using the 2+2 rule as previously described [27]; for progression to be documented, at least two new lesions had to be identified on the postdose 3 bone scan, and at least two additional lesions relative to the postdose 3 bone scan had to be identified on the postdose 6 bone scan.

### Statistics

All continuous variables were summarized by mean and standard deviation. Categorical variables were summarized by contingency tables with frequency and percentage. A swimmer plot was produced to depict changes in the responses and survival statuses of all participants throughout follow-up. All analyses were performed using SAS (version 9.4) and Prism 6 software. A trend test was performed to evaluate the association between survival and changes in γH2AX levels, baseline CTCs, declines in ALKP levels and the presence or absence of pain progression during the study period.

## Results

Ten patients were enrolled in the study between July 2015 and November 2016. The majority of patients had high Gleason score prostate cancer. Nine patients had received at least one prior therapy for CRPC, of whom six had received at least one regimen of cytotoxic chemotherapy, two had received sipuleucel-T, six received abiraterone and prednisone, and three received enzalutamide. All patients had at least ten bone metastases at baseline, and eight had ≥20 (Table 1).

**Table 1.** Baseline characteristics of patients and their responses to radium-223.

Patient number	1	2^†^	3	4	5	6	7	8	9^‡^	10
Age (years)	75	76	75	61	59	69	68	71	64	75
ECOG status	1	1	1	1	1	1	1	2	0	1
Gleason score	8 (4+4)	7 (3+4)	6 (3+3)	8 (4+4)	8 (4+4)	9 (5+4)	9 (5+4)	Unknown	9 (4+5)	7 (4+3)
Prior mCRPC therapy	None	Abi, Enza	Doce, Sip-T	Sip-T, Doce	Doce	Abi	Abi, Enza, Doce, Cabazi	Doce, Abi	Doce, Abi, Doce-Carbo, Enza	Abi, Doce, Cabazi
Bone metastases, no.	>20	>20	>20	>20	10–20	10–20	>20	>20	>20	>20
PSA, ng/ml	<20	>100	<20	20–100	20–100	20–100	>100	>100	>100	>100
AKLP, U/l	≥115	≥115	≥115	<115	<115	<115	≥115	≥115	≥115	≥115
Hemoglobin, g/dl	<12	≥12	<12	≥12	≥12	≥12	<12	<12	≥12	≥12
CTC count at baseline, no./7.5 ml^§^	1–5	>100	1–5	21–100	6–20	1–5	21–100	>100	6–20	>100
CTC count after dose 3, no./7.5 ml^§^	1–5	21–100	0	21–100	21–100	0	Incomplete	Incomplete	6–20	21–100
Post-treatment γH2AX increase^¶^	None	None	Dose 6	None	Dose 6	None	None	None	Dose 3	None
Post-treatment therapy	Doce	Doce, Olaparib	N/A	Abi	Enza, Doce-Carbo	Enza, Doce	Hospice	Hospice	Cabazi, Olaparib	Cabazi
Overall survival, months^#^	12.5	15.6	NR	13	16.5	NR	6	1.5	NR	6.3

† Subject with a germline mutation in BRCA2 D2723H (8395G>C).

‡ Subject with a germline mutation in PALB2 c.3113 G>A (p.Trp1038*).

§ 7.5 ml of peripheral blood used for CTC measurement.

¶ Defined as a 50% or more increase in the percent of CTCs positive for γH2AX (to at least 20% of CTCs), following at least one radium-223 treatment.

# As measured from the first dose of radium-223.

Abi: Abiraterone and prednisone; Cabazi: Cabazitaxel; Carbo: Carboplatin; CTC: Circulating tumor cell; Doce: Docetaxel; ECOG: Eastern Cooperative Oncology Group; Enza: Enzalutamide; mCRPC: Metastatic castration-resistant prostate cancer; N/A: Not Applicable; NR: Not reached; PSA: Prostate-specific antigen.

Eight patients received all six doses of radium-223. Two patients were taken off study after dose 1 because of rapidly progressive disease. All three patients with ≤5 CTCs at baseline were able to complete the full radium treatment course. The two patients with less than 5 baseline CTCs (patient 6 [ten bone lesions at baseline] and patient 3 [>20 bone lesions at baseline]) are currently alive, more than 30 and 38 months since undergoing radium-223 treatment, respectively (Table 1). Of note, these two patients were the only patients to undergo CTC conversion from detectable to 0 within 96 h (week 9), following dose three of radium-223. Baseline CTCs were not associated with the risk of death when evaluated as a binary variable with a cutoff of 5 (p = 0.095).

**Table 2.** Univariate analysis of overall survival using Cox regression analysis as well as Log-rank and a Trend test.

Variable	Level	Patients (no.)	Trend test, p-value
γH2AX	Increase	3	**0.035**
	No increase	7	
Baseline CTC count	≤5	3	0.095
	>5	7	
ALKP	Decline	4	**0.006**
	No decline	6	
Pain	No progression	6	0.056
	Progression	2	

Bold values indicate p < 0.05. CTC: Circulating tumor cell.

**Figure 2.**
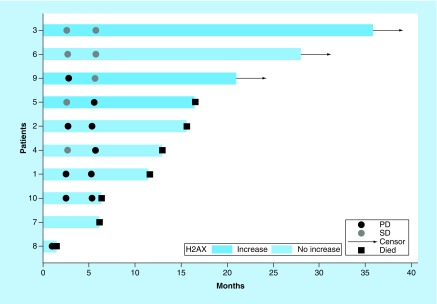
Swimmer plot detailing overall survival, disease progression as measured via bone scan imaging and the presence (shaded bar) or absence (blank bar) of a significant increase in γH2AX, defined as ≥50% increase in the percent of circulating tumor cells positive for γH2AX after at least one of the three measured radium-223 treatments. Patient 3 had a 68% increase in the percent of CTCs positive for γH2AX ([3 H2AX positive CTCs/3 CTCs]/[31 H2AX positive CTCs/52 CTCs]) after the sixth radium treatment. Patient 5 had a 56% increase in the percent of CTCs positive for γH2AX ([148 H2AX positive CTCs/318 CTCs]/[77 H2AX positive CTCs/258 CTCs]) after the sixth radium treatment. Patient 9 had a 167% increase in the percent of CTCs positive for γH2AX ([6 H2AX positive CTCs/9 CTCs]/[1 H2AX positive CTC/4 CTCs]) after the third radium treatment. The length of each bar represents the whole follow-up time for the corresponding patient. Patients 3, 6 and 9 had censored survival times. Black and gray circles indicate times of observing progression of disease and stable disease. CTC: Circulating tumor cell; PD: Progressive disease; SD: Stable disease.

As detailed in Figure 2, the percentage of CTCs that were positive for γH2AX significantly increased after at least one radium-223 treatment in three patients (3, 5 and 9). Two of these patients (3 and 9) are still alive, more than 38 and 23 months from their first dose of radium-223, respectively. Trend tests showed that patients with a significant increase in γH2AX had a lower risk of death (p = 0.035) (Table 2). Of note, all three of these patients had a tALKP decline of more than 30% at week 12. Although none of these three patients had a PSA response during radium-223 treatment, all three had a pain response to radium-223 as early as week 9.

Four patients (3, 4, 6 and 9) had a significant decline in ALKP during the study period. All four of these patients had at least stable bone imaging during the treatment period. Trend test results showed that patients with a significant decline in tALKP had a lower risk of death (p = 0.006). Two of the patients who received all six doses of radium-223 had pain progression during the treatment period. Pain progression during radium-223 therapy was not associated with poorer OS (p = 0.056). Two patients in our study had germline deleterious mutations in DNA homologous recombination repair deficiency (HRD): patient 2 had *BRCA2* D2723H (8395G>C) and patient 9 had *PALB2* c.3113 G>A (p.Trp1038*). Although neither patient had a PSA response, both had a decline in tALKP at week 9. Patients 2 and 9 had a CTC decline of 25 and 55%, respectively, but a CTC conversion to below 5 was not observed in either patient. An increase in γH2AX was observed in patient 9 but in not patient 2. The OS for patient 9 was at least 15 months longer than that of patient 2.

## Discussion

We have demonstrated the feasibility of performing interval assessments of both CTCs and γH2AX levels in mCRPC patients undergoing radium-223 treatment. In addition, we confirmed a prior report that demonstrated patients with ≤5 CTCs at baseline were able to complete a full course of radium-223 therapy [17]. Moreover, our CTC numeration data indicated a potential prognostic value of CTC conversion to 0 at week 9. Phase III trials testing abiraterone, enzalutamide, TAK 700 and cabozantinib in M1 CRPC have recently shown that CTC nonzero at baseline and 0 at week 13 is a response measure of prolonged survival [28]. Our study also confirmed the important association between declines in ALKP and superior survival outcomes [29–32]. Finally, increased γH2AX levels following radium-223 treatments were associated with a lower risk of death, warranting further investigation of γH2AX as a biomarker for patient response to radium-223 therapy.

The efficacy of radium-223 therapy for patients with HRD is of particular interest because of the theory that the double-strand DNA breaks it induces are more likely to go unrepaired in these patients [33]. A recent report based on a retrospective medical record review showed that patients with HRD who were treated with radium-223 were significantly more likely to have at least a 30% decrease in ALKP levels. In this study, patients in with HRD also had a significantly longer time to ALKP progression [34]. Although a decline in tALKP levels was observed in both patients (patients 2 and 9) with germline deleterious mutations in HRD, neither patient had CTC conversion after dose 3 at week 9. The CTCs of both patients increased by more than 50% after dose 6 with radium-223 compared with their baseline levels prior to radium-223 treatment (data not shown). Both patients received and responded to olaparib after radium-223. Patient 2 had a much greater disease burden than subject 9; the lack of increased γH2AX levels after radium-223 treatment in patient 2 could have contributed to their much shorter survival rate compared with patient 9. Both patients tolerated radium-223 well and had no grade 3 or 4 treatment-related adverse events.

The lack of robust clinical response to radium-223 in patients 2 and 9 could be attributed to the activation of alternative double-strand DNA repair pathways, including nonhomologous end joining. HRDs have important prognostic and predictive implications for patients with prostate cancer. Prostate cancer patients who have mutations in the DNA repair pathway (such as *BRCA1*, *BRCA2*, *ATM*, *RAD51D* and *PALB2*) have been shown to be significantly more likely to have advanced disease than patients without these mutations. Patients with these mutations have also demonstrated inferior OS [35–37]. Both the *BRCA2* and *PALB2* mutations are known to respond well to poly-ADP ribose polymerase (PARP) inhibitors [38]. PARP inhibitors ultimately lead to double-strand DNA breaks that cells with HRD are unable to repair [39]. PARP inhibitors are particularly effective in treating prostate cancer patients with HRD [38]. Androgen receptor inhibition has also been hypothesized to be a more effective treatment for prostate cancer patients with HRD because of the role that androgen receptor signaling plays in the regulation of DNA repair. However, studies have so far shown conflicting results regarding this correlation [40–42]. One case series has suggested that prostate cancer patients with *BRCA* mutations may be more responsive to the intrastrand cross-links affected by platinum-based chemotherapy [43,44].

Our study had several limitations. As our study was primarily exploratory, our analyses were limited by a small sample size; a larger study with a longer follow-up time is needed to further define the utility of γH2AX as a tumor marker in this setting. In addition, measuring both CTC and γH2AX levels is a relatively novel approach and may entail inherent inaccuracies. For example, the proper timing of γH2AX measurements are not clearly established in this setting. Moreover, the extent of an increase in γH2AX that is clinically significant is not yet established. Furthermore, as the assay checks CTCs for γH2AX positivity, it is possible that some CTCs may die before they are detected, thereby preventing the detection of changes in γH2AX. More generally, γH2AX may not be an ideal marker of the damage induced by radium-223 therapy. Some patients may have biologically less aggressive tumors and may do well even in the absence of an effective response to antineoplastic therapy. Finally, because measurements of pain are inherently subjective, associations with this measurement are by nature difficult to assess.

## Conclusion & future perspective

We have demonstrated that regular interval assessments of γH2AX are feasible for mCRPC patients receiving therapy with radium-223. We have also identified a possible association between changes in γH2AX and the risk of death among patients undergoing this therapy that could be further explored in a larger study with a longer follow-up. Given the limited number of patients exhibiting a PSA response to radium-223 and the current lack of biomarkers robust enough to predict a survival benefit from this treatment, the effective implementation of additional biomarkers could have significant clinical use [3,5,6]. Specifically, if further validated, measurements of γH2AX and CTCs could potentially be used to provide early information to clinicians regarding a patient’s response to radium-223 therapy, including the extent and duration.

**Summary points**

- On the basis of its survival benefit documented in the Phase III ALSYMPCA trial, radium-223 (Xofigo) is approved by the US FDA for the treatment of patients with symptomatic metastatic castration-resistant prostate cancer to the bone and no known visceral metastatic diseases.
- Only 16% of treated patients had a ≥30% decline in prostate-specific antigens at week 12 in the ALSYMPCA trial. Declines in total alkaline phosphatase or lactate dehydrogenase were associated with a longer overall survival (OS) but did not meet statistical surrogacy requirements for OS.
- We prospectively enrolled ten patients undergoing radium-223 treatment as standard of care and demonstrated that interval assessments of both circulating tumor cells and the DNA damage marker γH2AX were feasible. A possible correlation between increases in postradium-223 γH2AX-positive circulating tumor cells and longer OS was observed, which could warrant further evaluation.
